# Epicutaneous Sensitization to the Phytocannabinoid β-Caryophyllene Induces Pruritic Inflammation

**DOI:** 10.3390/ijms241814328

**Published:** 2023-09-20

**Authors:** Saadet Inan, Sara J. Ward, Citlalli T. Baltazar, Gabrielle A. Peruggia, Elham Javed, Ajay P. Nayak

**Affiliations:** 1 Department of Neural Sciences, Center for Substance Abuse, Lewis Katz School of Medicine at Temple University, Philadelphia, PA 19140, USA; saadet.inan@temple.edu (S.I.); sara.ward@temple.edu (S.J.W.); tuk93275@temple.edu (C.T.B.); 2 Department of Medicine, Center for Translational Medicine & Division of Pulmonary, Allergy and Critical Care Medicine, Jane and Leonard Korman Lung Center, Thomas Jefferson University, Philadelphia, PA 19107, USAelham.javed@jefferson.edu (E.J.)

**Keywords:** beta caryophyllene, dermatitis, pruritus, allergy, cannabis, terpene

## Abstract

In recent years, there has been increased accessibility to cannabis for recreational and medicinal use. Incidentally, there has been an increase in reports describing allergic reactions to cannabis including exacerbation of underlying asthma. Recently, multiple protein allergens were discovered in cannabis, yet these fail to explain allergic sensitization in many patients, particularly urticaria and angioedema. Cannabis has a rich chemical profile including cannabinoids and terpenes that possess immunomodulatory potential. We examined whether major cannabinoids of cannabis such as cannabidiol (CBD) and the bicyclic sesquiterpene beta-caryophyllene (β-CP) act as contact sensitizers. The repeated topical application of mice skin with β-CP at 10 mg/mL (50 µL) induced an itch response and dermatitis at 2 weeks in mice, which were sustained for the period of study. Histopathological analysis of skin tissues revealed significant edema and desquamation for β-CP at 10 mg/mL. For CBD and β-CP, we observed a dose-dependent increase in epidermal thickening with profound thickening observed for β-CP at 10 mg/mL. Significant trafficking of CD11b cells was observed in various compartments of the skin in response to treatment with β-CP in a concentration-dependent manner. Mast cell trafficking was restricted to β-CP (10 mg/mL). Mouse proteome profiler cytokine/chemokine array revealed upregulation of complement C5/5a (anaphylatoxin), soluble intracellular adhesion molecule-1 (sICAM-1) and IL-1 receptor antagonist (IL-1RA) in animals dosed with β-CP (10 mg/mL). Moreover, we observed a dose-dependent increase in serum IgE in animals dosed with β-CP. Treatment with β-CP (10 mg/mL) significantly reduced filaggrin expression, an indicator of barrier disruption. In contrast, treatment with CBD at all concentrations failed to evoke scratching and dermatitis in mice and did not result in increased serum IgE. Further, skin tissues were devoid of any remarkable features, although at 10 mg/mL CBD we did observe the accumulation of dermal CD11b cells in skin tissue sections. We also observed increased filaggrin staining in mice repeatedly dosed with CBD (10 mg/mL). Collectively, our studies indicate that repeated exposure to high concentrations of β-CP can induce dermatitis-like pathological outcomes in mice.

## 1. Introduction

Allergic reactions to *cannabis* have been reported over the last 5 decades [1,2,3]. These reports have been sporadic; however, in recent years, with increased access and decriminalization of *cannabis*, reactions to the plant have been frequently reported [2,4,5]. The symptoms of an allergic reaction to cannabis are very broad, but typically include contact (urticaria and angioedema) and respiratory (rhinoconjunctivitis, exacerbate underlying asthma) manifestations [3]. In rare cases, anaphylaxis has also been reported [6,7]. Studies published by our laboratory and elsewhere have reported type I hypersensitivity to cannabis proteins as a mechanism to explain allergic reactions to cannabis, with numerous allergens identified and subsequently validated [2,5,8,9,10]. More specifically, four cannabis allergens have been reported to date and include profilin (Can s 2) [10], non-specific lipid transfer protein (Can s 3) [5,8,11,12], oxygen-evolving enhancer protein (OEEP2 or Can s 4) [2,9] and Bet v 1 homologue (Can s 5) [10]. However, cases suggestive of allergic reactions to cannabis, particularly in occupational settings, do not yield detectable IgE antibodies to specific cannabis proteins [4]. However, there is a high prevalence of work-related allergic reactions (dermal and respiratory) among workers involved in growing and processing cannabis [13]. Most recently, the Occupational Safety and Health Administration (OSHA) conducted an investigation into a fatality in a cannabis grow facility which possibly occurred due to cannabis dust inhalation resulting in occupational asthma [14]. Thus, it is conceivable that other non-protein constituents of cannabis may contribute to observed reactions.

Cannabis is endowed with a rich chemical profile consisting of distinct cannabinoids, terpenes and terpenoids [15]. Cannabinoids are closely related chemical compounds that are naturally synthesized in the ‘buds’ of the female cannabis plant. Cannabinoids have been studied extensively for putative anti-microbial, immunomodulatory and psychoactive effects. Delta 9 tetrahydrocannabinol (Δ^9^-THC) is a major psychoactive component in cannabis with clearly established hallucinogenic and mood-altering abilities. Cannabidiol is the major non-psychoactive component produced by cannabis and is suggested to have anti-inflammatory capabilities. Moreover, cannabis consists of >100 other cannabinoids, many of them with an unclear physiological role. Detailed studies into these compounds have been limited owing to Schedule I restrictions (limiting accessibility to the plant) and complex pharmacological interactions. Finally, in addition to cannabinoids, cannabis also abundantly synthesizes other organic chemicals with poorly defined physiological effects.

In particular, terpenes have been previously reported as potent contact sensitizers, although the underlying mechanism has not been explored [16]. Terpenes are unsaturated hydrocarbon molecules commonly produced by many plants and chiefly impart unique flavor and aroma to the plant. Cannabis produces different terpenes including β-myrcene, α- and β-pinene, terpinolene, limonene and β-caryophyllene (β-CP) among others, which impart a unique aroma to a specific cannabis plant variety in various dominant combinations [15]. The volatile bicyclic sesquiterpene β-CP is produced abundantly by cannabis, constituting > 25% of the total terpene profile. Further, β-CP is also a cannabinoid receptor (CB2) agonist (full agonist), and synthetic and endogenous agonists of the CB2 receptor have been shown to contribute to dermal inflammation [17,18,19]. Thus, we hypothesized that β-CP could potentially drive allergic reactions in skin.

In the present study, we examined if β-CP can act as a contact sensitizer in a murine dermatitis model and contribute to pathophysiological features akin to urticaria and angioedema commonly observed in patients with allergic sensitization to cannabis. Specifically, we used an epicutaneous sensitization model to examine the capacity of β-CP to act as an inducer of contact dermatitis-like symptoms. Further, we examined the relative contribution of CB2-mediated mechanisms to affect these symptoms, since β-CP has been deemed as a selective agonist of CB2 receptor [20,21,22]. Moreover, since cannabidiol (CBD) is commonly used as a topical ointment, and renowned for its anti-inflammatory actions in different disease models [23,24,25], we examined its effects on the murine skin in this model as well.

## 2. Results

### 2.1. Epicutaneous Administration of β-CP Induces Itch-like Response in Mice

**Figure 1:**
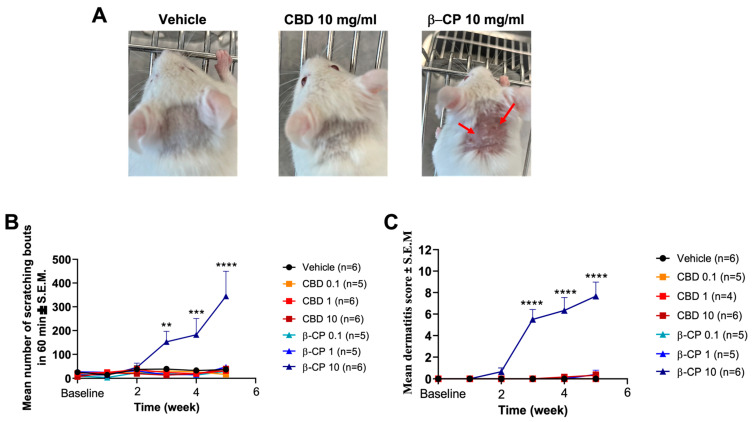
Repeated dosing with β-CP induces dermatitis-like features in mice. (**A**) Representative images of gross dermatitis in mice repeatedly dosed with vehicle, CBD (10 mg/mL) or β-CP (10 mg/mL). β-CP (10 mg/mL) induces significant edema, erythema and excoriation at the dosing site on the rostral neck (marked with red arrows). (**B**) Changes in the number of scratching bouts induced by CBD and β-CP. (**C**) Dermatitis scores (edema, erythema and excoriation) for repeated dosing with CBD and β-CP. (n = 4–6, two-way ANOVA followed by Tukey’s multiple comparison, ** *p* < 0.01, *** *p* < 0.001, **** *p* < 0.0001). All doses for CBD and β-CP are in (mg/mL).

Vehicle, CBD (all concentrations) and β-CP at 0.1 and 1 mg/mL did not induce scratching behavior and dermatitis (Figure 1). At the 2-week time point, for β-CP (10 mg/mL), we observed an increase in scratching bouts in mice (Figure 1A), along with clear signs of dermatitis including edema, erythema and excoriation (Figure 1B,C). These features progressed rapidly, were significant by the 3rd week and sustained over the course of the study.

### 2.2. Gross Histopathological Analysis Reveals Agonist-Dependent Effects

**Figure 2:**
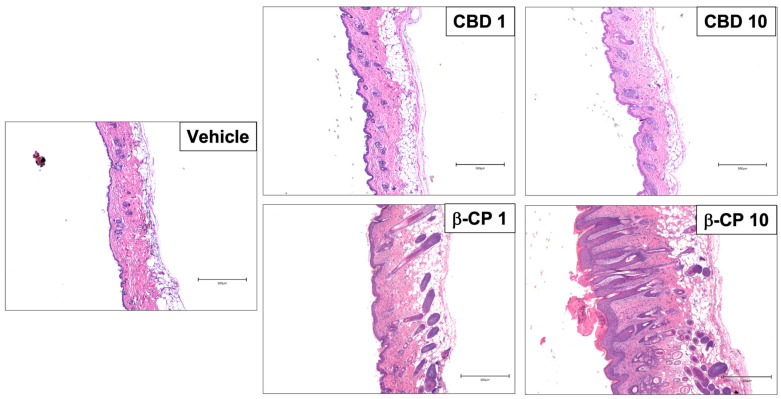
β-CP induces significant histopathological changes in a dose-dependent manner. Representative images of Hematoxylin and eosin (H&E) stained paraffin-embedded skin tissue sections from mice repeatedly dosed with vehicle, CBD (1–10 mg/mL) and β-CP (1–10 mg/mL). Data are collected from n = 3 animals for each treatment group. Scale bar 500 µm.

**Figure 3:**
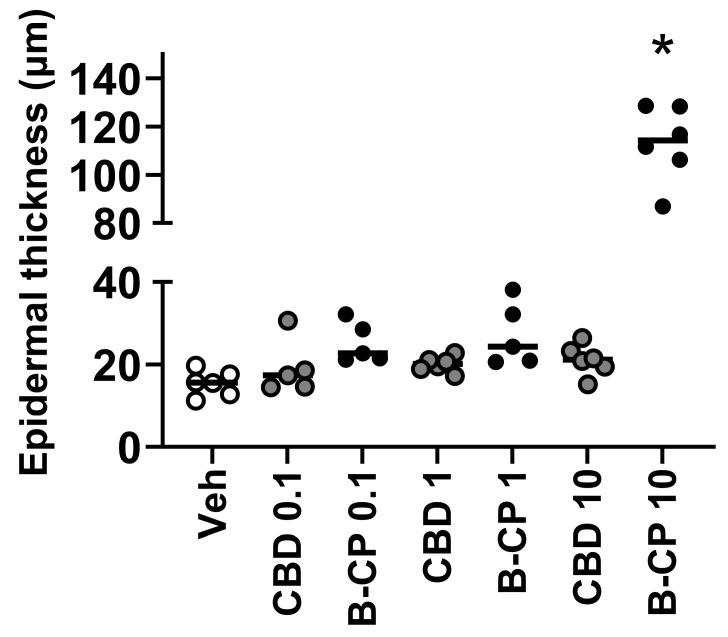
β-CP induces an increase in epidermal thickening in a dose-dependent manner. Mean values of epidermal thickness measurements from rostral neck tissue sections stained with H&E. Mice dosed with vehicle, CBD (1–10 mg/mL) and β-CP (1–10 mg/mL). Data are collected from n = 3 animals for each treatment group. * indicates *p* < 0.05.

To further detail the features of the dermatitis reaction to β-CP, we performed a microscopic examination of Hematoxylin and Eosin (H&E) stained sections. The stained sections revealed accumulation of cellular infiltrates in various compartments of the skin tissue following epicutaneous administration of β-CP in a dose-dependent manner, but not for cannabidiol (CBD) (Figure 2). Further, we observed significant diffused epidermal hyperplasia and elongation of rete ridges in the skin tissues from mice dosed with β-CP (10 mg/mL). Significant edema and the reorganization of hair follicles were also evident along with desquamation of the cornified layer of the skin. Finally, significant epidermal thickening was observed in tissue sections from mice dosed with β-CP (10 mg/mL). These inflammatory and remodeling features were absent in mice dosed with the same concentration of CBD (Figure 2 and Figure 3).

### 2.3. β-CP and CBD Induce Immune Cell Infiltration

**Figure 4:**
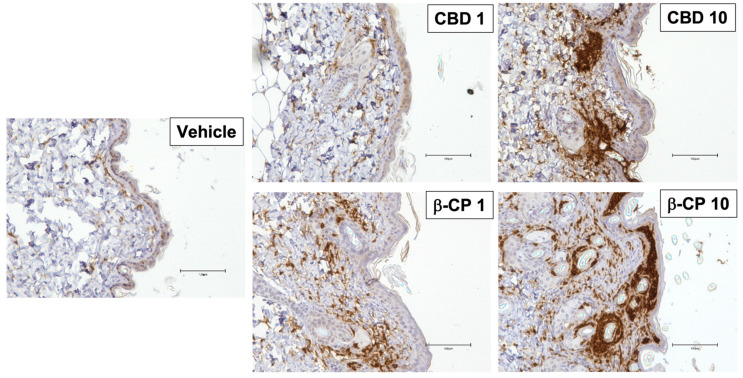
CBD and β-CP induce accumulation of immune cells in distinct compartments of skin tissue in a dose-dependent manner. Representative images of paraffin-embedded skin tissue sections stained for CD11b using immunohistochemistry approach. Mice dosed with vehicle, CBD (1–10 mg/mL) and β-CP (1–10 mg/mL). Data are collected from n = 3 animals for each treatment group. Scale bar 100 µm.

Since we observed the accumulation of cellular infiltrates in skin tissues in response to β-CP, we next examined if these cells expressed CD11b (integrin alpha M), a leukocyte marker expressed on neutrophils [26], and antigen-presenting cells including dermal macrophages and certain dermal dendritic cells [27]. CD11b^+^ cells were identified in skin tissue sections using immunohistochemistry approach (Figure 4). In the vehicle-treated mice, we observed very few CD11b^+^ cells in the dermis. Treatment with β-CP resulted in a dose-dependent increase in the recruitment of CD11b^+^ immune cells, with profound accumulation of cells at β-CP (10 mg/mL). CD11b^+^ cells are very prominently observed in the epidermal compartment and surrounding hair follicles. Trafficking CD11b^+^ cells were also observed in the dermis. Interestingly, CBD treatment did not evoke the significant accumulation of CD11b^+^ cells at 0.1 and 1 mg/mL concentrations. Surprisingly, at CBD (10 mg/mL), there was significant recruitment of CD11b^+^ cells in the dermis and around hair follicles; however, unlike β-CP, there was no evidence of invasion of the epidermis.

**Figure 5:**
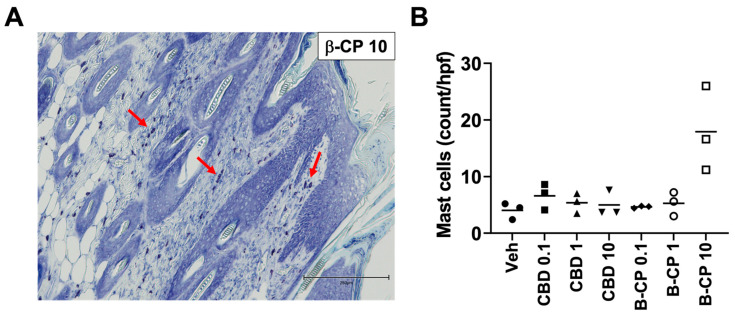
β-CP induces accumulation of mast cells in dermis in a dose-dependent manner. (**A**) Representative image of paraffin-embedded skin tissue section (β-CP 10 mg/mL) stained with Toluidine blue for staining mast cells (indicated by red arrows) using immunohistochemistry approach. Scale bar 250 µm. (**B**) Quantitative assessment of mast cell recruitment in skin tissues from animals dosed with vehicle, CBD (0.11–10 mg/mL) and β-CP (0.1–10 mg/mL). Data are represented as mast cells (counts/hpf) collected from n = 3 animals for each treatment group. Hpf = high-power field.

Next, we examined if treatment with β-CP (10 mg/mL) also recruited mast cells to the inflamed skin. Using toluidine blue (a metachromatic stain), we detected purple-stained cells indicative of mast cells in the dermis of mice treated with β-CP (10 mg/mL) (Figure 5A,B). Across all other treatment groups, we observed minimal mast cell numbers over the baseline vehicle treatment conditions (Figure 5B).

### 2.4. β-CP and CBD Differentially Activate Soluble Immune Components

**Figure 6:**
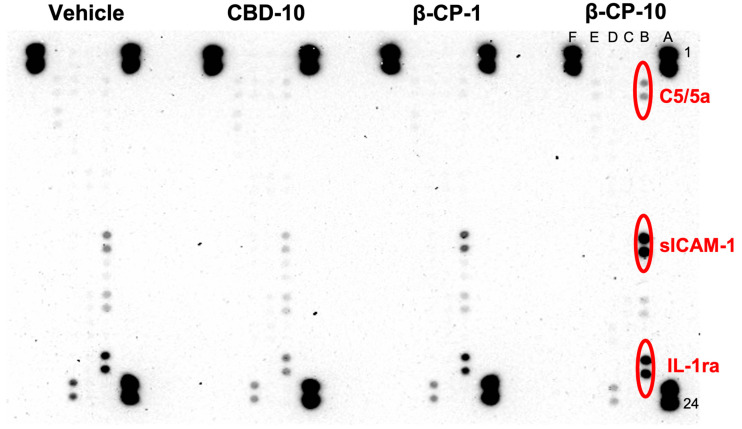
Skin tissue Chemokine/cytokine protein levels. Nitrocellulose membranes are shown for neck tissue for vehicle CBD-10, β-CP 1 and 10 treatments. A1, 2, 23, 24, F1, 2 are reference spots, and F23, 24 are negative control. Each membrane was used for one group. For each group, cytokine and chemokine levels were measured in pooled samples (5–6 mice). In total, 300 μg samples for each group were used. Sample/antibody/streptavidine-HRP complex was measured using chemiluminescent detection reagents. Light is produced at each spot in proportion to the amount of cytokine bound. Luminescence was quantitated by a Fuji Digital camera using imageGauge^®^ software (Version 4.1). Results were stated as Arbitrary Units. C5/5a, sICAM-1, and IL-1ra were found to have increased in mice treated with β-CP 10.

Next, we investigated the predominant cytokine/chemokine signals associated with the cellular trafficking into skin compartments following treatment with CBD and β-CP. A higher luminescence of Complement C5/5a (anaphylatoxin), the soluble intracellular adhesion molecule-1 (sICAM-1) and the interleukin 1 receptor antagonist (IL-1RA) were detected in skin samples of the β-CP (10 mg/mL) compared to vehicle, CBD (10 mg/mL) and β-CP (1 mg/mL) (Figure 6). The average duplicated readings as arbitrary units (AU) for C5/5a were 4966.5, 5818, 4168 and 20,684 for vehicle, CBD (10 mg/mL), β-CP (1 mg/mL) and β-CP (10 mg/mL), respectively. Almost a fourfold increase in AU unit was detected in β-CP (10 mg/mL) from vehicle. AUs for sICAM-1 were 22,723 for vehicle, 9316 for CBD (10 mg/mL), 32,230 for β-CP (1 mg/mL) and 190,165 for β-CP (10 mg/mL). While β-CP (10 mg/mL) induced almost an eightfold increase in sICAM-1, a threefold reduction was found in the CBD (10 mg/mL) treatment group. AUs for IL-1Ra were 59,505 for vehicle, 22,356 for CBD (10 mg/mL), 46,666 for β-CP (1 mg/mL), and 107,208 for β-CP (10 mg/mL). Similarly, while the CBD treatment reduced IL-1Ra, treatment with β-CP (10 mg/mL) almost twofold increased the IL-1Ra expression. There were no other detectable changes in the rest of the chemokine/cytokine levels following treatments.

### 2.5. β-CP and CBD Evoke Distinct Patterns of Filaggrin Expression

**Figure 7:**
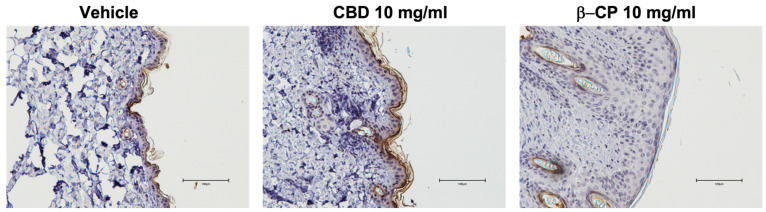
Differential effects of CBD and β-CP on filaggrin expression. Representative image panel of paraffin-embedded skin tissue section from mice repeatedly dosed with vehicle, CBD (10 mg/mL) and β-CP (10 mg/mL) were immunostained for filaggrin using immunohistochemistry approach. Representative data are collected from n = 3 animals for each treatment group. Scale bar 100 µm.

The dermal barrier is essential for maintaining homeostasis in the skin. Since β-CP at a high concentration induces significant inflammation and remodeling in the skin, we sought to examine the objective indices of barrier integrity. Filaggrin is an epidermal (specifically stratum corneum) skin protein that is involved in aggregating keratin filaments for maintenance of skin barrier function and protection from pathogens, allergens and dehydration [28]. Loss in filaggrin (either by environmental insults or genetic loss of function mutations) is typically associated with skin inflammation including atopic dermatitis. In this study, we examined the expression of filaggrin in skin tissue following repeated treatment with CBD and β-CP using immunohistochemistry approach. While animals treated with vehicle showed normal expression of filaggrin in the stratum corneum, treatment with β-CP resulted in significant depletion of filaggrin in murine skin tissues (Figure 7 left and right panels). Interestingly, filaggrin expression was observably higher in mice treated repeatedly with CBD (Figure 7 middle panel).

### 2.6. β-CP and CBD Differentially Stimulate IgE Accumulation in Serum

**Figure 8:**
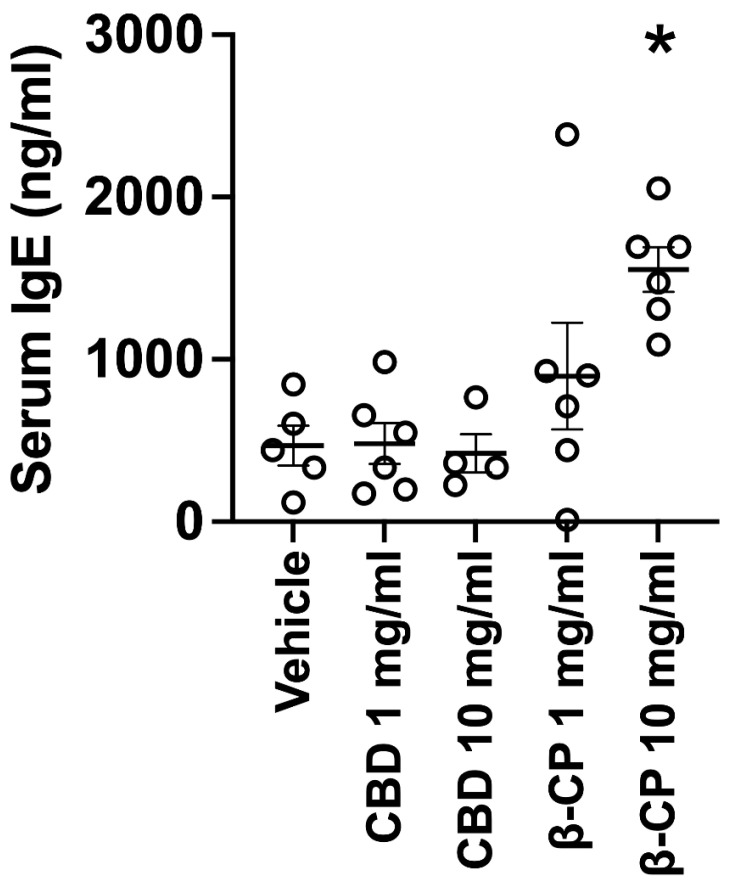
Differential effects of CBD and β-CP on circulating IgE. Mean values of total IgE from serum samples of mice dosed with vehicle, CBD (1–10 mg/mL) and β-CP (1–10 mg/mL). Data are collected from n = 4–6 animals for each treatment group. * indicates *p* < 0.05.

To further detail the nature of dermatitis/pruritic inflammation, we examined if repeated dosing with β-CP (and CBD) resulted in the expansion of the pro-allergic immunoglobulin IgE. Specifically, we observed a dose-dependent increase in total IgE in serum from mice repeatedly dosed with β-CP, with a significant increase at β-CP (10 mg/mL) (Figure 8). In contrast, either dose of CBD (1–10 mg/mL) did not yield any change in serum IgE and were comparable to mice treated with vehicle.

## 3. Discussion

Allergic sensitization to cannabis is an emerging public health issue. While preliminary studies by our group and others have identified type I hypersensitivity, they fail to explain the symptomology observed in all cannabis-sensitized individuals [2,4]. Notably, urticaria and angioedema are commonly observed in cases of a suspected cannabis allergy, but only half of these indicate the presence of IgE to cannabis-specific proteins, suggesting a role for other cannabis components. Our studies detailed herein indicate that the cannabinoid sesquiterpene β-CP acts as a contact sensitizer and possibly mediates hypersensitivity reactions to the cannabis plant.

**Figure 9:**
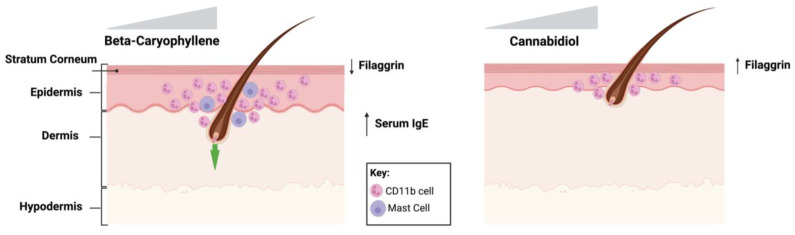
An illustration to summarize the differential effects of repeated exposure to β-CP and CBD. β-CP induces pruritic inflammation indicative of dermatitis in a dose-dependent manner. β-CP exposure results in significant recruitment of immune cells and reorganization in the skin compartments. Specifically, we observed immune cell trafficking into the epidermis with flaking of the stratum corneum and recessed hair follicles in the dermis. Further, mast cells were commonly noted in the inflamed skin tissue of mice dosed with β-CP along with an increase in circulating IgE in serum. In contrast, CBD exposure does not produce pruritic lesions in the skin and does not result in an increase in serum IgE. However, exposure to CBD does result in the recruitment of CD11b immune cells to the epidermis which may play a role in promoting anti-inflammatory actions in the skin. Green arrow indicates internalization of the hair follicle.

In the present study, we demonstrate that chronic topical exposure to β-CP, but not to CBD induces contact dermatitis in mice. Dermatitis was determined by counting scratching bouts combined with the scoring of physical features of edema, excoriation, and erythema. These findings were further substantiated by the inclusion of data from microscopy-based approaches that detail the physical changes in skin compartments. Mice developed dermatitis features by the third week of dosing with β-CP. Histopathologic changes included significant epidermal thickening, presence of CD11b^+^ immune cells in the epidermis, increase in mast cells, increase in chemokines C5/5a, sICAM-1 and IL-1ra, increased serum total IgE and compromised epidermal integrity (Figure 9). Results of the present study clearly indicate that chronic exposure of β-CP may result in an allergic reaction that manifests as a pruritic lesion at the site of exposure.

Cannabis is endowed with a rich chemical profile of diverse compounds capable of modulating human physiological systems through complex mechanisms. While significant resources have been dedicated towards understanding the pharmacological, physiological and pathophysiological attributes of THC and cannabidiol; other components of cannabis have poorly defined physiological roles. One such group of compounds are terpenes which are aromatic-ring-containing compounds and are commonly used in fragrances. Terpenes (α-pinene, β-pinene, limonene, terpinolene, β-myrcene and β-caryophyllene) are abundantly produced in cannabis [15]. Among these, the bicyclic sesquiterpene β-caryophyllene (β-CP) is abundantly produced by cannabis plants [15], is extremely volatile and lipophilic. Further, studies have shown that β-CP possesses anti-inflammatory [20,29], neuroprotective [30,31,32], antiproliferative [33] and antidepressant [34] functions. Finally, terpenes such as β-CP may also be involved in the ‘entourage effect’ which potentiates the physiological actions of cannabinoids including endocannabinoids [35]. Thus, strains with high levels of β-CP may be of interest for medicinal and recreational use.

However, terpenes (particularly in essential oils and fragrances) have been suggested to contribute to contact allergy and dermatitis although mechanistic insights are severely lacking [36,37,38]. Certain physical and chemical features of terpenes may contribute to their allergenicity. Firstly, terpenes impart a sticky and gluey physical property to cannabis buds, which may allow them to persist on the skin for long durations [15]. Secondly, terpenes are extremely lipophilic resulting in enhanced transdermal delivery across the skin barrier [39]. One mechanism by which β-CP may exert its allergenic effects is through its auto-oxidation on contact with air. Terpenes have been previously suggested to contribute to allergic sensitization on oxidation [16,37,40]. β-CP is also a full agonist of the CB2 receptor [20]. While the stimulation of the CB2 receptor is commonly linked with anti-inflammatory outcomes, the oxidation of β-CP or its interactions with host proteins can alter activity at the receptor site [21]. Further, the significant recruitment of immune cells occurs to the different skin compartments, including hair follicles that could be the primary site of immune activation to β-CP. Indeed, previously we showed that toluidine diisocyanate (TDI; volatile and lipophilic) on exposure to skin, partitions to hair follicles thus allowing a critical site for immune sampling by distinct epidermal and dermal dendritic cell populations [41]. Interestingly, CBD also induced comparable recruitment of immune cells around the hair follicles, although we did not observe any overt signs of pruritus in these animals, indicating CBD is not immunologically inert, and the type of immune cells recruited by CBD and β-CP and need phenotyping. Indeed, our studies demonstrate that unlike CBD, β-CP uniquely induces the recruitment of mast cells in the dermis. Another possibility is that repeated dosing of β-CP may induce an itch response and inflammation that is sustained by endocannabinoids such as 2-AG, as previously shown for oxazolone [19]. Additional studies are needed to examine how phytocannabinoids and terpenes modulate the endocannabinoid-mediated regulation of skin homeostasis.

Among other immunological mediators, we observed a fourfold increase in C5/5a, an eightfold increase in sICAM-1, and a twofold increase in IL-1RA following β-CP (10 mg/mL) in skin samples. On the other hand, CBD (10 mg/mL) induced reductions in both sICAM and IL-1RA. C5a, the fifth component of C5, is an anaphylatoxin and possesses leukocyte chemotactic activities. Intradermal administration of C5a to volunteers induced wheal and flare reactions with pruritus in a dose-dependent manner as well as more potent than histamine, compound 48/80 and morphine [42]. Mast cell degranulation, endothelial cell edema and leucocyte infiltration were found in the skin biopsies of the volunteers in the same study. The activation of coagulation factors and complement systems C3 and C5 have been studied on development of chronic spontaneous urticaria and it was suggested that coagulation factors and C5/C5a induce histamine release from mast cells [43]. In both pediatric and adult atopic dermatitis and eczema patients, as well as in chronic spontaneous urticaria blood sICAM levels were found to be increased [44,45,46]. No evidence has been reported involvement of IL-1Ra in contact or atopic dermatitis so far. However, results of our studies clearly show that chronic exposure to β-CP results in increase in C5a and sICAM that can induce histamine release from the mast cells, at least during the ‘effector phase’ of the response to β-CP. A decrease in those cytokines following CBD exposure possibly suggests a mechanistic basis for anti-inflammatory effects of CBD. Longitudinal studies that allow dissection of the dynamic changes in cytokine/chemokines in the ‘sensitization phase’ will be essential to detail the sequential events that link β-CP to dermatitis. Nevertheless, our results unequivocally establish the immunological mechanisms involved in β-CP-mediated dermatitis. In our previous study [47], using dinitrofluorobenzene (DNFB) chronic exposure-induced contact dermatitis, we identified unique immunological signatures. Since different chemokine and cytokine activation was found in two different chemically induced dermatitis, it indicates that the pathogenesis of β-CP-elicited dermatitis is different from DNFB-induced dermatitis.

β-CP and CBD also demonstrated significant differences in their ability to alter the epidermal barrier. While β-CP significantly depleted filaggrin protein in the stratum corneum (an indicator of barrier integrity), CBD potentiated filaggrin expression thus likely enhancing epidermal barrier function. CBD and its utility as a skin protective agent have been commonly noted in the literature [48,49]; however, the specific mechanisms have been unclear. In this study, we demonstrate that the potentiation of filaggrin and the improvement of the skin barrier function is a likely mechanism for the therapeutic actions of CBD in skin diseases.

In summary, our studies using the murine model of dermatitis demonstrate that β-CP can act as a contact sensitizer and may be responsible for allergic contact dermatitis-like presentation in symptomatic individuals. The translational potential of our findings in the animal model is currently unclear. However, a few key observations underscore the significance of our findings. Our model relies on direct epicutaneous inoculation of β-CP in animals, which is comparable to the direct handling of cannabis among recreational users, where we overwhelmingly observe urticaria and hives in contact with the plant. Our findings are also relevant for exposures in occupational settings where volatile and lipophilic β-CP can accumulate on the skin during a typical work period. Our observations also suggest that β-CP and CBD may differentially activate immune cells and endocannabinoid systems and is currently the subject of our ongoing studies. It is likely that while some allergic reactions are explained by type I hypersensitivity reactions to specific cannabis protein allergens, others are likely caused by IgE to terpenes (especially β-CP) and possibly also involving type IV hypersensitivity mechanisms. It remains unclear as to why not all individuals demonstrate allergic reactions to cannabis on contact/exposure, which may depend on levels of these terpenes in individual strains, the nature of primary exposure (recreational vs. occupational) and other host-specific factors (atopy, susceptibility to skin sensitization, etc.). Indeed, other terpenes may demonstrate similar potential for driving allergic reactions and will be the subject of our studies in the future. Since cannabis strains can be distinguished based on unique terpene profiles [15], it will be interesting to examine if certain variations have a higher propensity to cause allergic reactions.

## 4. Materials and Methods

### 4.1. Methods

#### 4.1.1. Cannabinoids, Antibodies, and Other Reagents

β-caryophyllene (β-CP) was purchased from Sigma Aldrich (St. Louis, MO, USA). Cannabidiol (CBD; derived from hemp) was obtained by Cayman Chemicals (Ann Arbor, MI, USA). Rabbit polyclonal anti-filaggrin and recombinant rabbit monoclonal anti-CD11b (clone EPR1344) antibodies was purchased from AbCam (Cambridge, UK).

#### 4.1.2. Animals

Male Swiss Webster mice (Taconic Biosciences, Germantown, NY, USA) weighing 23–25 g were housed in a temperature- and humidity-controlled environment with a 12 h light–dark cycle. Animals were supplied food and water *ad libitum*. All endpoints associated with behavioral testing was collected between 11:00 a.m. and 5:00 p.m. All experimental procedures were approved by the Institutional Animal Care and Use Committee of Temple University and conducted according to the NIH Guide for the Care and Use of Laboratory Animals.

#### 4.1.3. Epicutaneous Sensitization Model and Behavioral Analyses

**Figure 10:**
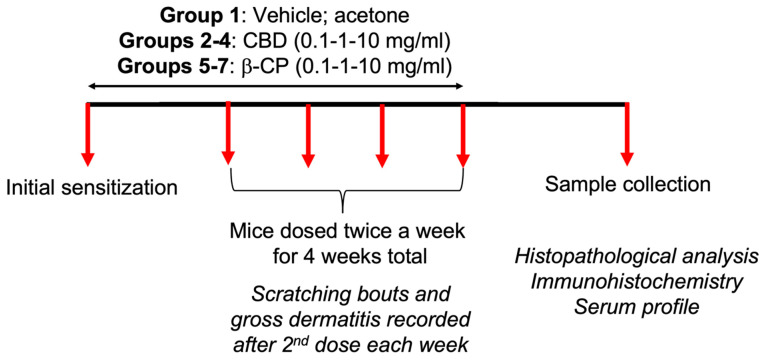
Dosing schedule for CBD and β-CP. Swiss Webster mice (n = 4–6) were randomly assigned to different experimental groups and were observed for the establishment of baseline scratching bouts and dermatitis. Subsequently, mice were sensitized with the application of different doses of CBD and β-CP (0.1–1–10 mg/mL) to the abdominal region. One week later, mice were repeatedly dosed on rostral neck with CBD and β-CP for an additional four weeks, and scratching bouts and gross dermatitis features were recorded after the 2nd dose each week. On the conclusion of dosing, mice were euthanized for sample collection and downstream analyses. One group of mice were sensitized and repeatedly challenged with vehicle (acetone) and served as control animals.

Epicutaneous sensitization with CBD or β-CP was performed using approaches described previously [47]. Briefly, mice were grouped into 7 different dosing schemes (vehicle, acetone; CBD 0.1–1–10 mg/mL; β-CP 0.1–1–10 mg/mL). Two days prior to the application of compounds, the fur from the dorsal region of the neck and the abdomen (small part) was shaved to expose the underlying skin. Mice were dosed using the dosing scheme described in Figure 10. Briefly, mice were assigned to each dosing group randomly and observed for 1 h to establish baseline scratching bouts. Following that, 100 μL of compounds (vehicle, CBD or β-CP) was applied to the abdominal area of the skin for initial sensitization. A week later, topical application was made to the skin of the rostral neck 2 times a week with 50 μL of the compounds for 5 weeks. Once a week, 24 h following the 2nd application of each compound, scratching bouts were counted for 1 h. At the end of the 5th week evaluation, mice were euthanized via exposure to CO_2_ followed by cervical dislocation and skin samples were obtained from each animal. Samples were kept at −80 °C until use.

#### 4.1.4. Examination of Dermatitis

Following the completion of the dosing schedule, mice were examined for gross pathological changes to the dorsal neck skin. Dermatitis was scored on a scale of none (0), mild (1), moderate (2) and severe (3) for pathological features of (a) erythema/hemorrhage, (b) edema, (c) excoriation/erosion and (d) dryness. Scores were aggregated from each dermatitis characteristic with the maximal possible score of 12. Dermatitis was evaluated once a week prior to the quantification of scratching bouts.

#### 4.1.5. Skin Tissue Lysates and Measurement of Chemokine and Cytokine Levels

Tissue lysates were generated by excising the dorsal skin on the neck from each mouse and subsequently homogenizing in phosphate-buffered saline (PBS)-containing protease inhibitors. Triton X-100 (1% final conc.) was added to tissue homogenate and samples were frozen at −80 °C, before being thawed and centrifuged at 10,000× *g* for 5 min. The cellular debris was discarded, and the supernatant was stored at −80 °C for downstream analyses. The total protein concentration in each tissue lysate sample was measured using the NanoDrop 2000 spectrophotometer (Thermo Fisher, Waltham, MA, USA) using standard approaches.

The relative expression of 40 different cytokines/chemokines in skin tissue lysates was determined using a proteome profiler mouse cytokine array kit (R&D Systems Inc., Minneapolis, MN, USA) as detailed previously [47]. Three hundred micrograms of pooled tissue lysate samples from mice of each experimental group were applied directly onto the proteome profiler array nitrocellulose membrane, which was pre-coated with antibodies for each analyte in duplicates. Antibody interactions with specific cytokines/chemokines were complexed with streptavidin-horse radish peroxidase (HRP) and subsequently developed using chemiluminescence approach. Chemiluminescent signal emanating from each spot was detected using a Fuji Digital camera and quantitative measurements were performed using the imageGauge^®^ software (Version 4.1). Results were reported as arbitrary units (A.U.) for each sample normalized to corresponding signal from the vehicle control group.

#### 4.1.6. Histopathological Analysis and Immunohistochemistry

Murine dorsal skin tissue treated with different agents was excised and skin tissue samples were fixed in 10% formalin buffered saline prior to tissue processing for histology at the end of 5th week evaluations. Paraffin-embedded skin sections were sectioned and stained with Hematoxylin and Eosin (H&E) for histological analysis using methods described previously [50,51] and assessed to evaluate the extent of inflammation and determine epidermal thickening owing to repeated exposure to CBD and β-CP. The quantification of epidermal thickening was performed using methods described previously [41]. Briefly, we imaged H&E-stained skin tissue sample slides using the EVOS M7000 microscope (Invitrogen, Thermo Fisher Scientific, Waltham, MA, USA). Images were captured using a camera and processed using EVOS Analysis software (V 2.0.2094.0, Invitrogen, Thermo Fisher Scientific). Epidermal and dermal thickness was measured using a calibrated measurement tool interfaced with the imaging software (V 2.0.2094.0) using methods described previously [52,53]. Measurements were obtained from 10 fields along the length of the skin tissue section at high magnification. Measurements were obtained for n = 4–6 animals per treatment group.

Paraffin-embedded rostral neck skin tissue sections were stained for CD11b and filaggrin using standard immunohistochemistry approaches. Briefly, paraffin-embedded blocks were sectioned at 4 μm. The slides were deparaffinized in Shandon Varistain Gemini ES Autostainer. Antigen retrieval was performed with DAKO PT-Link using Tris-EDTA Buffer (pH 9.0) at 98 °C for a total time of 20 min. Immunohistochemistry (IHC) staining was performed using an intelliPATH FLX^®^ Automated Slide Stainer (Biocare medical, LLC, Pacheco, CA, USA). Primary antibodies: CD11b (Abcam, Cat#: ab133357, 1:5000), Filaggrin (Abcam, Cat#: ab81468, 1:200). Primary antibodies were incubated at room temperature for 30 min. Biotinylated anti-Rabbit (Vector Laboratories, Newark CA, USA, cat#: BA-1000) secondary antibody and ABC-HRP complexes (Vector Laboratories, Cat#: PK6100) were applied following the primary antibodies with 30 min incubation each reagent at room temperature. Three 1xTBST washes were performed between each step above. The signals were visualized using 3′,3′ Diaminobenzidine (DAB) substrate (Vector Laboratories, Cat#: SK-4103-400). Slides were then washed with deionized water and proceeded with Hematoxylin counter stain, dehydration and clearing in Shandon Varistain Gemini ES Autostainer and mounted with Permount Mounting Medium in Clearvue Automatic Coverslipper.

Paraffin-embedded rostral neck skin tissue sections were also stained with the metachromatic Giemsa stain/Toluidine blue to examine the degranulation of infiltrating mast cells using standard methods. Mast cells were identified based on characteristic staining features and quantified from 5 high magnification fields of the tissue section.

### 4.2. Serum IgE Analysis

Mouse serum total IgE measures were established using a mouse immunoglobulin IgE single plex assay (Eve Technologies, Calgary, AB, Canada) using the Millipore Luminex platform interfaced with Bio-Plex Manager software (Millipore, Burlington, MA, USA).

### 4.3. Data Analysis and Statistics

All datasets are presented as means ± standard error of means (SEM). The statistical analysis and significance between different treatment groups for scratching bouts and dermatitis scores was determined using two-way ANOVA followed by Tukey’s multiple comparison and for serum IgE using Ordinary one-way ANOVA followed by Sidak’s multiple comparisons; *p* < 0.05 was accepted as significant. All statistical analyses were performed using the Prism 9 software (GraphPad, San Diego, CA, USA).

## Data Availability

All data is stored in secure computer drives at Thomas Jefferson University and Temple University and will be made available at request.
